# A classification of human resource management bundles for the inclusion of vulnerable workers

**DOI:** 10.3233/WOR-230314

**Published:** 2024-09-11

**Authors:** Amber Kersten, Marianne van Woerkom, Goedele Geuskens, Roland Blonk

**Affiliations:** a Department of Human Resource Studies, School of Social and Behavioural Sciences, Tilburg University, Tilburg, The Netherlands; b Healthy Living, Netherlands Organisation for Applied Scientific Research (TNO), Leiden, The Netherlands; c Optentia Research Focus Area, North-West University, Vanderbijlpark, South Africa

**Keywords:** Rehabilitation, diversity, equity, inclusion, vulnerable populations, latent class analysis

## Abstract

**BACKGROUND:**

Despite the societal importance to improve understanding of the role of employers in the inclusion of workers with a distance to the labor market, scant knowledge is available on the effectiveness of human resource management (HRM) bundles for the inclusion of vulnerable workers.

**OBJECTIVE:**

This paper studies which HRM bundles are applied by employers that hired people with a distance to the labor market, and to what extent these different bundles of HRM practices are related to employment of workers with specific vulnerabilities, such as people with disabilities or people with a migration background.

**METHODS:**

A latent class analysis of 1,665 inclusive employers was used to identify HRM bundles based on seven HRM practices: financial support practices, specialized recruitment, promotion and career opportunities, training opportunities, part-time work, job crafting, and adaptations to the workplace.

**RESULTS:**

Six bundles were identified: a recruitment and development bundle (34.4% of employers), a development bundle (24.8%), maintenance-focused practices (16.5%), a recruitment bundle (9.4%), a sustainable employment bundle (8.9%), and passive HRM (6.0%). *Post-hoc* analyses showed the probability of hiring specific vulnerable groups for each bundle (e.g., sustainable employment bundles showed the highest overall probability to hire people with a physical disability).

**CONCLUSION:**

Nuancing what is suggested in strategic HRM literature, we conclude that both extensive HRM and focused HRM bundles can be successful for the employment of vulnerable workers. In conclusion, there is no one-size-fits-all approach to inclusive employment and employers, large or small, can tailor their HRM systems to include vulnerable workers.

## 1 Introduction

The changes on the labor market related to globalization, financial crises, and the COVID-19 pandemic, have globally worsened the position of various minorities on the labor market. These so-called vulnerable workers, such as people with a migration background, people with disabilities, people who are long-term unemployed or people with a low-educated background, face increasing exclusion from stable employment [e.g., 1– 4]. Resulting from this persistent exclusion from employment, these individuals may experience an increasing distance to the labor market, referring to the seemingly lengthy and difficult path between their (temporary) unemployment and the (re-)entry into the world of work. As the vulnerability of these workers is growing and labor shortages persist, there is a need for improving the rehabilitation and inclusion of vulnerable groups on the labor market [5].

Whereas in the past, the inclusion of vulnerable groups was primarily addressed by economic and public policies, more recent studies have investigated the employer’s role in promoting the inclusion of vulnerable workers [6, 7]. The involvement of employers is thought to be key, since their organizational practices and policies contribute to inclusive workplaces that offer opportunities for the rehabilitation of vulnerable groups on the labor market [8].

Given the complexity of creating inclusive workplaces, Strategic Human Resource Management (SHRM) literature suggests that bundling HRM practices may help to create powerful HRM approaches (i.e., HRM bundles) resulting in long-term inclusion of vulnerable groups [9], whereas the use of single HRM practices, such as recruitment, may not [10– 12]. Still, most of the literature on HRM bundles and their effectiveness has primarily focused on mainstream or performance-driven practices (e.g., recruitment practices aimed at a general working population, or performance management practices), whereas bundles of specialized practices with direct implications for the inclusion of vulnerable workers (e.g., job crafting, work adaptations) have been overlooked [13].

For this reason, and as a next step to further unravel this complex problem, we aim to investigate to what extent inclusive employers apply and bundle mainstream and inclusive HRM practices, such as training, specialized recruitment, promotion opportunities, or work adaptations, to create inclusive workplaces for vulnerable workers, and to what extent these HRM bundles are related to organizational characteristics and the employment of specific vulnerable groups. To this end, we use latent class analysis (LCA) to answer the following research questions:

*RQ1*. Which types of inclusive HRM bundles can be identified based on the HRM practices that are applied by inclusive employers, who have hired people with a distance to the labor market in the past two years? 

*RQ2.* To what extent do organizations that apply these HRM bundles differ in their organizational characteristics and the hiring of specific vulnerable groups? 

In our study we focus on employers, who have hired at least one worker with a distance to the labor market in the past two years. Studying the behaviors of these inclusive employers can help to generate relevant insights into labor market inclusion and improve the development of policies that are targeted at all employers, including those that strive to hire vulnerable groups [14]. By doing so, we contribute to the current literature on how labor market inclusion can be achieved through a demand-side approach [e.g., 10, 15].

In addition, we focus on actual employer behavior instead of motives or attitudes. By doing so, we respond to the criticism that previous literature has focused too much on employers’ intentions [16], attitudes [17] or motives for inclusive behavior [18, 19], which have found to be unreliable predictors of actual behavior [14]. In addition, previous research shows that the perceptions of employers’ intention to be inclusive differ considerably between different organizational actors [20]. By applying a LCA, this study uses a theoretically founded, yet data-driven approach to classify HRM practices and bundles that inclusive employers apply. This provides a more reliable approximation of the *actual* behavior of inclusive employers and how the employers differ from each other, compared to previous studies that focused solely on *intended* employer behavior.

Additionally, we provide these insights not solely for the more extensively covered group of people with disabilities, but also for minorities which are often overlooked in strategic HRM literature [21]. Examples of such overlooked groups are individuals with a migration background or refugee status, or individuals who have lower levels of education (e.g., exclusively primary education) as a result of limited access to or opportunity for education, for instance due to a learning disability.

### 1.1 Inclusive HRM practices and bundles for vulnerable workers

Within the field of strategic HRM research, it is highlighted that HRM practices should be applied and studied in their configuration with other HRM practices (i.e., HRM bundles). The logic behind this is that if HRM practices are combined into a larger, aligning HRM bundle, they will have more impact than the sum of each HRM practice individually (i.e., 1 + 1 = 3). To illustrate, an HRM bundle that combines training based on teamwork with a reward system related to teamwork will strengthen to positive effect of each of these individual HRM practices into a more efficient bundle [22]. In line with this logic of 1 + 1 = 3, the meta-analysis of Subramony [23] indeed shows the increased effects of bundles of HRM practices compared to individual practices in terms of retention, and operational, financial and overall performance in general working population. Research that focused on diversity and equality management has also shown that the bundling of inclusive HRM practices increases the effectiveness of diversity management [12]. However, previous HRM literature has primarily focused on mainstream HRM practices, whereas specialized and inclusive HRM practices designed for vulnerable workers have not been given much attention thus far [13]. Therefore, more insight is needed into the effects of bundles of both mainstream and specialized HRM practices [11]. In addition, previous research has mainly addressed organizational outcomes related to performance or turnover [23], whereas outcomes related to the labor market inclusion of vulnerable workers are largely overlooked. Based on the literature, the following HRM practices should be considered when exploring HRM bundles aimed at the inclusion of vulnerable workers.

First, based on a previous study by Napathorn [24], the use of *recruitment practices* as a part of inclusive HRM bundles was shown to be important to include vulnerable workers. Based on interviews with social entrepreneurs in Thailand, Napathorn [24] conceptualized a recruitment focused HRM bundle. This recruitment bundle moved beyond the more commonly used mainstream recruitment practices within most organizations, and included practices related to the recruitment of workers through word-of-mouth or employee referrals. These practices helped the social enterprises to facilitate low turnover and to hire candidates that aligned with the available position and the goal of the social enterprise. Other studies highlight a similar importance of specialized recruitment practices for vulnerable workers [11]. Examples of such recruitment practices aimed at vulnerable workers are job creation or reshoring, with a focus on creating jobs for vulnerable groups [25] or collaborating with external parties in recruitment [26]. Hence, specialized recruitment practices seem to be an important aspect of inclusive HRM bundles aimed at vulnerable workers.

Second, two previous studies on inclusion of older workers and social entrepreneurship show support for HRM bundles related to offering training, promotion, and career opportunities [24, 27] to ensure that sustainable employability of minorities is achieved [28, 29]. Kooij and colleagues [27] furthermore distinguish between a maintenance focus and a development focus. The first is aimed at maintaining the current level of functioning within the organization, by offering regular training, whereas the latter focuses on growth within the organization, by complementing training with growth opportunities. Therefore, it seems to be important to account for practices related to both *training*, which may be aimed at primarily maintaining existing skills, and development, which may be aimed more at *promotion* within the organization and the gaining of new skills, when forming inclusive HRM bundles for vulnerable workers.

Third, literature on HRM bundles aimed at older workers distinguished bundles aimed at accommodating workers by reducing work demands and stimulating sustainable employment [27]. Examples of practices within this approach are offering part-time work possibilities, facilitating job crafting (i.e., employee-initiated changes to work content or context), and increasing workplace support by using adaptations or modifications of the work (employer-initiated) [16, 30– 33]. Hence, we expect that accommodative practices related to part-time work opportunities, job crafting, and work adaptions form a principal element of HRM bundles for vulnerable workers, since they allow for tailor-made solutions that fit to the needs of diverse groups of vulnerable workers.

Financial support practices may provide financial means for the organization to acquire and offer additional support to the workers with certain vulnerabilities, such as work adaptations or an extended contract [34]. These financial support measures may not only contribute positively to the individual employee but also help to alleviate the pressure on the employer by generating the financial means for accommodations [35]. Therefore, financial support practices are thought to form a relevant part of the inclusive HRM bundles aimed at vulnerable workers.

To conclude, previous literature has identified several HRM practices that play a vital role in the creation of inclusive workplaces. In the current study, we will focus on the following HRM practices: specialized recruitment, training opportunities, development opportunities, part-time work opportunities, job crafting, work adaptations and financial support practices. We will empirically examine the bundling of these HRM practices by employers that recently hired a vulnerable worker.

## 2 Methods

### 2.1 Design and procedure

To identify different HRM bundles for the rehabilitation of people with a distance to the labor market, we used the data of the Netherlands Employers Work Survey in 2019 (NEWS) [36]. NEWS is a large-scale study within the Netherlands that focuses on employment arrangements within organizations on the Dutch labor market, which employ at least two people. The data collection of this research was designed in accordance with the Dutch Ministry of Social Affairs and Employment and based on informed consent of participants. To generate a nationally representative sample, stratified sampling was applied based on 41 sectors and five organizational size classes, which were selected from the Netherlands National Job Information System. Organizations were contacted with an announcement letter and a follow-up call or email. Respondents were mostly owners of the company or (HR) managers, as these organizational representatives are most knowledgeable about the (HR) practices that are applied within the organization. Both digital and paper-and-pencil questionnaires were available.

### 2.2 Sample

In total 23.914 organizations were approached to participate in NEWS-2019. Of these 23.914 organization, 13.645 complied with the selection criterium of at least two employees. In total 9.222 organizations did not participate. This resulted in a final sample of 4.423 organizations and a response rate of 32 percent. Since we focus on employers that are actively involved with vulnerable groups within their organization, additional selection criteria were used to generate the final sample. Firstly, only organizations were selected that indicated to have knowingly hired a person with a distance to the labor market in the past two years. This resulted in a sample of 1.676 employers. Secondly, employers that offer ‘sheltered employment’ were filtered out of the final sample, since these employers are profiled as profoundly different from the largest share of the labor market [37]. This resulted in the final dataset of 1,665 employers.

Of this final sample, 64.8 percent of the organizations were based in the profit sector, 28.8 percent were based in the non-profit sector, and 6.4 percent performed both profit and non-profit activities. The average number of employees at the organizations was 230.04 and approximately 59.8% of the organizations indicated to be a part of a larger organization (Dutch or international). Of all respondents, 25.5 percent was director or owner of the organization, 52.1 percent was HR manager, 10.9 percent was a general manager and 11.5 percent had another job (e.g., supervisor or office manager).

### 2.3 Measures

**The use of specialized recruitment practices** was measured with one dichotomous item, which combined the responses of the respondent on six separate items (0 = none of the practices were applied, 1 = one or multiple practices were applied). The use of one combined item instead of six separate items was used to meet the LCA requirements of binary data. The separate items asked respondents if their organization applied (or concretely initiated) one or more of the following recruitment practices, specifically aimed at hiring workers with a distance to the labor market: 1) job creation based on new jobs; 2) job creation based on existing jobs (e.g., renewal of old jobs); 3) offering work experience to vulnerable groups (e.g., temporary jobs, internships); 4) temporary external hiring (through an employment agency); 5) reshoring work; or 6) collaborations with other employers. These items were based on previous research on specialized recruitment of vulnerable groups through job creation and reshoring [25], external collaborations [26], and temporary work [38].

**Training opportunities** were measured with one dichotomous item asking if the organization offered training or educational opportunities to their personnel (0 = practice was not applied, 1 = practice was applied).

**Promotion and career opportunities** were measured with one dichotomous item asking if the organization offered any promotion or career opportunities to their personnel (0 = practice was not applied, 1 = practice was applied).

**Parttime work opportunities** were measured with one dichotomous item asking if the organization offered their employees the opportunity to work part-time (0 = practice was not applied, 1 = practice was applied).

**Job crafting** was measured with one dichotomous item asking the respondent if job crafting was applied within their organization, to ensure that (vulnerable) employees could be sustainably employed (0 = practice was not applied, 1 = practice was applied).

**Work adaptations** were measured with one dichotomous item asking the respondents if work adaptations (i.e., broadening or changing job requirements) were applied within their organization, to ensure that (vulnerable) employees could be sustainably employed (0 = practice was not applied, 1 = practice was applied).

**The use of financial support** was measured with one dichotomous item that combined the responses on four separate items, which asked if the respondent used any of the financial practices, in line with the financial arrangements offered on the Dutch labor market (0 = none of the practices were applied, 1 = one or multiple practices were applied) [39]. Again, a combined item instead of separate items was used to meet the requirements of the LCA. Respondents were asked in four separate items if their organization used one or more of the following four financial support measures to hire vulnerable groups: 1) subsidy for adaptation of the workplace; 2) wage dispensation; 3) premium discount; or 4) wage cost subsidy.

To explore which groups of vulnerable workers were employed by organizations using different HRM bundles, additional items were included. *The hiring of specific vulnerable groups* was measured using six dichotomous items asking respondents to indicate whether they employed at least one worker of the following groups: people with a cognitive disability, psychologically vulnerable groups, people with a physical disability, people with a lower level of education or people with a learning disability, long-term unemployed groups, refugees or workers with a migration background.

*Organizational characteristics* included questions related to sector (1 = profit, 2 = non-profit sector, 3 = (semi-) public sector), number of employees (0 =≤100 employees, 1 = > 100 employees), whether vulnerable workers were mentioned in the organization’s mission statement (0 = no, 1 = yes), whether the organization was part of a larger organization (0 = no, 1 = yes), growth of revenues in the last two years (0 = no, 1 = yes) and growth of profits in the past two years (0 = no, 1 = yes).

### 2.4 Analysis

LCA using Latent GOLD 6.0 was conducted. LCA strives to classify similar respondents into groups and specify the number and form of these groups [40]. In this sense, LCA allows us to identify latent classes based on observed variables. The LC model is specified as:

$$\begin{array}{cc} & P\left(y_{1},y_{2},y_{3},y_{4}\right)\\ & =\sum_{c=1}^{C}P\left(X=c\right)P\left(y_{1},y_{2},y_{3},y_{4}|X=c\right) \end{array}$$

*P* (*X* = *c*) represents the probability of a respondent belonging to a certain class *c* and *P* (*y*_1_, *y*_2,_ *y*_3_, *y*_4_|*X* = *c*) represents the probability of this respondent having a response pattern given their class membership to class *c*. The overall probability for the population of respondents is calculated as a weighted average of class membership *P* (*y*_1_, *y*_2,_ *y*_3_, *y*_4_|*X* = *c*); using the weight of class proportions *P* (*X* = *c*). To compare different models, the bootstrap *p* of the likelihood-ratio chi-squared goodness-of-fit test statistic *L*^2^ was used. In addition, the Bayesian information criterion (BIC) was considered, as this information criterion is thought to be superior to other statistics, such as Akaike information criterion (AIC). The BIC is sample size adjusted and has shown to be particularly useful in larger samples, like the sample in this study [41, 42]. A Bootstrap three-step approach to LCA was applied [42]. In the first step the latent class model was selected, based on relevant fit measures. In the second step, class membership of each employer was determined by considering the highest probability. The result of this step is a categorical variable, which allows comparison of the different classes on relevant characteristics. The third and last step was focused on estimating the probability of hiring specific vulnerable groups, given the class membership. Wald chi-square *post-hoc* analyses were conducted to determine between-class differences.

## 3 Results

### 3.1 Model selection

Table 1 demonstrates the fit statistics for the estimated models ranging from a one-class model to a ten-class model. The optimal model was selected based on several statistical criteria. The Bayesian Information Criterion (BIC) was lowest for the six-class model, indicating the optimal solution. The bootstrap *p*-value of this model was 0.984, indicating excellent model fit. In addition, the six-class model demonstrated sufficient latent class separation, with an entropy R^2^ of 0.715. Based on these fit statistics and interpretation of the clustering, the six-class model was selected as the optimal representation of the data, meaning that six different bundles of organizational practices were distinguished. This means that the complexity of the model was reduced from the 128 potential combinations of HRM practices (*potential combinations* = 2*^*k*^*, with k representing the number of indicators in the model, in this case 2^7^ = 128) to six dominant bundles of HRM practices.

**Table 1:** Model fit evaluation for LCA models of inclusive HRM bundles

Model	LL	BIC	Npar	L^2^	*df*	Class. Err.	Entropy R^2^
1-Class	– 5,943.23	11,938.38	7	1,153.42	120	0.000	1
2-Class	– 5,689.02	11,489.30	15	645.01	112	0.054	0.716
3-Class	– 5,616.23	11,403.07	23	499.43	104	0.042	0.842
4-Class	– 5,564.12	11,358.18	31	395.20	96	0.129	0.708
5-Class	– 5,496.50	11,282.29	39	259.97	88	0.055	0.875
*6-Class*	– *5,462.78*	*11,274.19*	*47*	*192.54*	*80*	*0.169*	*0.715*
7-Class	– 5,440.52	11,289.00	55	148.00	72	0.167	0.722
8-Class	– 5,425.58	11,318.47	63	118.12	64	0.164	0.743
9-Class	– 5,413.54	11,353.73	71	94.04	56	0.220	0.686
10-Class	– 5,399.86	11,385.71	79	66.69	48	0.230	0.688

Note. LL (Log-Likelihood), BIC (Bayesian Information Criterion), Npar (Number of Parameters), L^2^ (Chi-Squared Test Statistic), *df* (Degrees of Freedom), Class. Err. (Classification Error). The selected model is highlighted in italics.

### 3.2 Class prevalence

The results presented in Table 2 and Supplementary Figure 1 show the item-response profiles for the six classes, indicating the probability that an organization belonging to a specific class applied (a combination of) the HRM practices. This table shows that class 1 and 5 combined different HRM practices into extensive HRM bundles, whereas class 2 and 4 applied more focused HRM bundles with a restricted number of practices and class 3 even opted for only two practices. Lastly, class 6 shows a low probability of applying any practices. We will describe the application of practices for each of the classes below.

**Table 2:** Item-response profile for the 6-Class model of inclusive HRM bundles

Classes					
	1	2	3	4	5	6
Label of class	Recruitment and Development HRM bundle	Development HRM bundle	Maintenance HRM practices	Recruitment HRM bundle	Sustainable Employment HRM bundle	Passive HRM
% of total sample	34.4	24.8	16.5	9.4	8.9	6.0
Indicator
Financial support	0.585	0.304	0.404	0.671	0.323	0.011
Recruitment practices	0.953	0.227	0.133	0.950	0.743	0.142
Promotion and career opportunities	0.938	0.999	0.176	0.008	0.977	0.003
Training opportunities	0.990	0.938	1.000	0.497	1.000	0.005
Parttime work	0.978	0.878	0.914	0.749	0.805	0.482
Job crafting	0.151	0.019	0.251	0.000	0.974	0.330
Work Adaptations	0.025	0.134	0.000	0.003	0.595	0.027

Note. Probabilities over.5 (i.e., a larger probability of applying the practice, compared to *not* applying the practice) are highlighted in grey.

The first class (*Recruitment and Development HRM bundle*) represented 34.4% of the employers. This bundle showed a high probability of applying specialized recruitment, training, parttime work, promotion and career opportunities and the use of financial support. The practices that these employers applied are aimed at both the recruitment as well as development of vulnerable workers and are therefore indicative of a combination of two HRM approaches, labeled as recruitment and development.

The second class (*Development HRM bundle)* represented 24.8% of the sample and was characterized by a high probability of offering development focused HRM practices: training opportunities, and promotion and career opportunities. In addition, these employers showed a high probability of offering part-time work opportunities.

The third class (*Maintenance HRM practices*) represented 16.5% of the sample and showed a high probability of offering training opportunities and part-time work. In contrast to the development HRM bundles, this bundle showed a low probability of offering promotion and career opportunities. Therefore, the primary aim of this HRM approach is on maintenance rather than development.

The fourth class (*Recruitment HRM bundle*) consisted of 9.4% of the sample. This class showed a high probability of applying specialized recruitment, in particular temporary employment, using financial support, and offering part-time work. Based on this combination of financial and recruitment practices, this class is labelled as the recruitment focused HRM bundle.

The fifth class (*Sustainable Employment HRM bundle*) consisted of 8.9% of the sample and showed a high probability of facilitating job crafting and work adaptation to ensure sustainable employment. Furthermore, this class was characterized by bundling specialized recruitment, promotion and career opportunities, training opportunities, and part-time work. This bundle is aimed at providing means for sustainable employment (in terms of job crafting and work adaptations), specifically aimed at vulnerable workers.

The sixth and last class (*Passive HR*) consisted of 6.0% of the sample. Although these employers have hired vulnerable workers in the past two years, the probability of applying any of the HRM practices was low, making this class passive compared to the other classes in the sample.

### 3.3 Employer characteristics and probability of hiring specific groups

To gain more insights into the different HRM bundles among inclusive employers, *post-hoc* analyses were conducted, and the characteristics and cross-group differences are presented in Table 3. Table 4 shows significant cross-group differences for the prediction of the hiring of specific vulnerable groups. Detailed Chi-Square statistics of the significant differences in superscript in Table 4, are available upon request. Below, we first paint the picture of each HRM bundle by showing the results on organizational characteristics and specific groups hired for each cluster. Afterwards, we provide a general reflection.

**Table 3:** Mean score comparison on characteristics

	Classes						
	1	2	3	4	5	6
	Recruitment and development HRM bundle	Development HRM bundle	Maintenance HRM practices	Recruitment HRM bundle	Sustainable employment HRM bundle	Passive HRM	Sample
**Organizational characteristics (0 = no, 1 = yes)**
> 100 employees	0.49 ^2,3,6^	0.28 ^1,4^	0.32^,1,4^	0.53 ^2,3,6^	0.42 ^6^	0.08 ^1,4,5^	0.43
Part of a larger organization	0.50 ^2^	0.36 ^1,4^	0.50	0.60 ^2,6^	0.51	0.28 ^4^	0.48
Vulnerable groups in mission	0.59 ^2,3,6^	0.24 ^1,3,4,5^	0.44^1,2^	0.61 ^2,6^	0.50 ^2^	0.28 ^1,4^	0.50
Profit sector	0.62 ^2^	0.73 ^1,4^	0.71	0.55 ^2,6^	0.65	0.88 ^4^	0.65
Non-profit sector	0.06 ^2,6^	0.08 ^1,4^	0.06	0.06 ^2,6^	0.08	0.08 ^1,4^	0.06
(Semi-)public	0.32	0.19	0.23	0.39	0.27	0.04	0.29
Growing revenue in past 2 years	0.44	0.44	0.41	0.46	0.50	0.48	0.44
Growing profits in past 2 years	0.53	0.52	0.51	0.57	0.67	0.52	0.54
**Financial support (0 = no, 1 = yes)**
Subsidy adaptations workplace	0.16 ^2^	0.06 ^1,4^	0.13	0.20 ^2^	0.12	0.00	0.13
Wage dispensation	0.39 ^2,6^	0.17 ^1,3,4^	0.40 ^2,6^	0.33 ^2,6^	0.27	0.00 ^1,3,4^	0.32
Premium discount	0.34 ^2,6^	0.14 ^1,3,4^	0.41^2,5,6^	0.40 ^2,5,6^	0.21 ^3,4^	0.00 ^1,3,4^	0.30
Wage cost subsidy	0.30 ^2,3,6^	0.15 ^1,3,4^	0.44 ^1,2,5,6^	0.32 ^2,6^	0.17 ^3^	0.00 ^1,3,4^	0.27
**Specialized recruitment (0 = no, 1 = yes)**
Job creation	0.23 ^2,6^	0.00 ^1,3,4,5^	0.19 ^2^	0.32 ^2,6^	0.18 ^2,6^	0.00 ^1,4,5^	0.18
Renewing old jobs	0.21 ^2,4,6^	0.00 ^1,3,4,5^	0.19 ^2,4^	0.36 ^1,2,3,5,6^	0.15 ^2,4^	0.00 ^1,4^	0.17
Work experience jobs	0.85 ^2,5,6^	0.00 ^1,3,4,5^	0.82 ^2,5,6^	0.87 ^2,5,6^	0.56 ^1,2,3,4,6^	0.00 ^1,3,4,5^	0.64
Hiring external workers	0.25 ^2,6^	0.00 ^1,3,4,5^	0.27 ^2,6^	0.32 ^2,5,6^	0.17 ^2,4^	0.00 ^1,3,4^	0.19
Reshoring work	0.01	0.00	0.01	0.03	0.00	0.00	0.01
Collaborations with local parties	0.08	0.00	0.03	0.13	0.06	0.04	0.06

Note. Although clusters may show a small probability to apply certain practices in Table 2, results may show a low mean of application of these practices in Table 3, due to our use of so-called modal clustering [41], meaning that only participants that did not apply certain practices could be assigned to a certain cluster, which explains some unexpectedly low values in Table 3; Significant cross-group differences (*p* < 0.05) are indicated in superscript, using the class number that the class significantly differed from.

**Table 4:** Probabilities and cross-group differences given class membership

	Classes					
	1	2	3	4	5	6
	Recruitment and development HRM bundle	Development HRM bundle	Maintenance HRM practices	Recruitment HRM bundle	Sustainable employment HRM bundle	Passive HRM
Vulnerable group hired
People with a cognitive disability	0.447 ^2,3,4^	0.131 ^1,5,6^	0.079 ^1,5,6^	0.194 ^1,6^	0.364 ^2,3^	0.402 ^2,3,4^
People who are psychologically vulnerable	0.421 ^3,4,6^	0.352 ^4,6^	0.279 ^1,6^	0.168 ^1,2,5^	0.442 ^4,6^	0.089 ^1,2,3,5^
People with a physical disability	0.242 ^4^	0.262 ^4^	0.282 ^4^	0.083 ^1,2,3,5^	0.327 ^4^	0.226
People with a learning disability or low education	0.498 ^2,3^	0.203 ^1,4^	0.110 ^1,4,5,6^	0.460 ^2,3^	0.354 ^3^	0.356 ^3^
People that are long-term unemployed	0.225 ^2,3,4,5^	0.449 ^1^	0.481 ^1^	0.491 ^1^	0.572 ^1^	0.341
People with a migration background and/or refugee	0.172 ^2,5,6^	0.039 ^1,4,5,6^	0.130 ^5,6^	0.255 ^2^	0.388 ^1,2,3^	0.358^2,3^

Note. Significant cross-group differences (*p* < 0.05) are indicated in superscript, using the class number that the class significantly differed from.

Employers with a *recruitment and development HRM bundle* were significantly larger compared to other employers and were mostly profit sector organizations. These employers more often mentioned vulnerable groups in their mission and scored higher on the use of financial practices and specialized recruitment. Compared to others, they showed the highest probability to hire people with a learning disability, low-educated people, or people with a cognitive disability.

Employers who used a *development HRM bundle* employed over 100 employees or were part of a larger organization less often. They were mainly profit-sector employers, who scored lower on mentioning vulnerable groups in their mission statement. These employers scored low on the use financial practices and specialized recruitment. These employers mostly hired long-term unemployed or psychologically vulnerable workers. Compared to other classes, they showed lower probabilities of hiring people with a cognitive disability, learning disability, or people with a migration background and refugees.

Employers that exclusively applied *maintenance HRM practices* less frequently employed over 100 employees or were part of a larger organization. Compared to development focused HRM bundles, these employers more often mentioned vulnerable groups in their mission statement. These employers more often applied financial practices (e.g., wage cost subsidies) and specific recruitment practices (e.g., work experience jobs). In contrast to several others, employers that applied maintenance HRM practices significantly more often hired long-term unemployed workers.

Organizations that applied the *recruitment HRM bundle* more often employed over 100 employees or were part of a larger organization and were mostly profit or mixed sector organizations. These employers more often used financial practices (e.g., premium discount) and more often hired temporary workers or offered work experience jobs. This class showed a high probability of hiring long-term unemployed workers and people with a learning disability and a low probability of hiring people with physical disabilities and psychologically vulnerable groups.

The employers that applied *sustainable employment HRM bundles* often mentioned vulnerable groups in their mission statement. In addition, they made less use of financial practices, but made more use of recruitment practices (e.g., hiring external wor-kers). The most hired groups were long-term unemployed workers, psychologically vulnerable workers and people with a migration background or refugees. The probability of hiring people with a migration background or refugees was even highest of all.

*Passive HRM* was least often applied by larger organizations and were most often applied by profit sector organizations. These employers least often mentioned vulnerable groups in their mission statement compared to the other clusters and scored lower on the use of financial or recruitment practices. This class most often hired people with a cognitive disability, learning disability and people with a migration background or refugees. Compared to others, this class showed the lowest probability of hiring psychologically vulnerable groups.

To summarize, the results indicate that the most extensive HRM bundles (class 1 and 5) were more common among larger employers. For instance, employers who applied the recruitment and development HRM bundle consisted significantly more often of over 100 employees compared to those, who solely applied the development or maintenance approaches. Also, the more extensive HRM bundles were most common among organizations that had an inclusive mission statement. Lastly, results showed that the development HRM bundle, and passive HRM were significantly more often applied in the profit sector, aligning with a more economic rationality that may be common in this sector. Surprisingly, no significant differences were found for revenue growth or profit growth between organizations with different HRM bundles.

## 4 Discussion

This study adds to our understanding of inclusive employer behavior by showing that inclusive employers are a diverse group that differ significantly from each other in the application of HRM bundles. In specific, our results show support for six distinct HRM bundles: a recruitment and development HRM bundle, a development HRM bundle, maintenance HRM practices, a recruitment HRM bundle, a sustainable employment HRM bundle, and a passive HRM bundle. The HRM bundles identified show that inclusive employers strongly differ in their use of HRM practices, ranging from applying extensive bundles of HRM practices to a more focused application of single HRM practices or even passive approaches.

These findings nuance previous HRM literature on the general working population that suggest that extensive bundling approaches are more effective compared to the application of single HRM practices [e.g., 9– 11], by demonstrating that more specified HRM approaches may contribute to hiring of vulnerable workers equally compared to extensive bundles. Results showed that the extensive HRM bundles (e.g., sustainable employment HRM bundle), which were mainly applied by larger organizations with probably more broadly developed HR department [43], were related with the highest probability to hire the widest scope of groups of vulnerable workers. Still, smaller organizations with narrow or even passive HRM approaches showed a significantly higher chance to hire certain specific vulnerable worker groups (e.g., people with a cognitive disability, people with a migration background, or refugees), compared to organizations that applied extensive HRM bundles. This finding shows that small organizations may have an advantage over bigger organizations due to their flexible nature and more informal workplace practices, which allow for more tailormade solutions. Therefore, these results call into question the necessity of extensive HRM systems for the inclusion of vulnerable workers. Further, our findings call for future research that takes into account the number of vulnerable workers hired and that investigates the potential relevance of factors beyond HRM bundles for the work participation of vulnerable workers, such as factors relating to the (informality of the) work environment [e.g., 44].

Additionally, whereas previous research has suggested that employers may hesitate to hire vulnerable workers due to concerns about finances related to hiring a vulnerable worker [e.g., 45], our results do not show a relation between recent revenue or profits and the application of HRM practices. In fact, 44.1% of the inclusive employers in our sample indicated that they had no growing profits during the time in which they hired a vulnerable worker and 53.7% indicated that they had no growth in their revenue during this time. Still, this did not stop these employers from hiring a person with a distance to the labor market. There may be several explanations for this. Firstly, as our results show, the financial support practices offered by the Dutch government are used by a substantial share of the sample. Previous research has shown that tax breaks and salary subsidies are seen as helpful strategies for improving the hiring and retention of workers with disabilities [45]. Therefore, despite potential (biased beliefs about) financial risks, organizations may be open to invest in the inclusion of vulnerable workers once they use tax breaks or subsidies. In addition to this, there may be other positive drivers for inclusive employer behavior that outweigh potential financial risks. A recent systematic review on employers hiring people with disabilities showed that pro-social motivations to hire vulnerable workers, expecting competitive advantage, and a strong belief in the unique advantages of hiring certain vulnerable groups, may be important positive drivers of inclusive behavior [46].

Among the applied HRM practices within our study population, training opportunities and parttime work were the HRM practices that were mostly commonly used. This aligns with previous research that shows that employers value both these practices for sustaining the inclusion of vulnerable groups, as they allow the vulnerable worker to gain relevant skills and ensure flexibility to make the work accessible for vulnerable workers [28, 29]. The least used HRM practices were work adaptations and job crafting. The reason may be that these practices often require complex, customized solutions, and impact the organization of work, which may be simply too much of an investment for most employers. Future research is needed to study to what extent biased beliefs about lacking job-person fit play a role [35], to what extent job crafting interventions may be beneficial to help craft jobs to vulnerable employee’s needs [47], and how employers can be supported to apply such inclusive HRM practices.

In addition to these findings, our study uncovers relationships between the HRM bundles that are applied and the groups that are hired. For instance, the sustainable employment bundle, which is characterized by job crafting and work adaptations, showed the highest probability to include a wide variety of workers, such as people with physical limitations, people with a migration background, or psychologically vulnerable workers. This suggests that if employers adapt the work(place), they can facilitate employment for a wide range of vulnerable groups. Furthermore, the focused bundles (aimed at either recruitment, maintenance, or development), seem to result primarily in the inclusion of long-term unemployed persons. These bundles may be especially important to update the skills and work experience of long-term unemployed persons. These insights may serve as a guideline for organizations that target specific vulnerable groups.

### 4.1 Practical implications

Our results may support HR professionals by providing a benchmark of the HRM bundles used by employers. The findings emphasize that there is no one-size-fits-all approach to inclusion. Not only extensive HRM bundles, as commonly seen among larger organizations, but also focused HRM bundles, which may be more appropriate for smaller organizations, may be successful in realizing the employment of vulnerable workers. This provides a more nuanced image of inclusive workplace practices and highlights that extensive HRM systems may not be necessary to create inclusive workplaces. This is especially important as previous research has highlighted that one of the main reasons why employers do not hire vulnerable workers, is that they feel that they are unsure about the needs of vulnerable workers and fear that they do not have the right or too little resources to support the employment of vulnerable workers [44].

With these findings, we hope to inspire more employers to find ways to contribute to labor market inclusion. Based on our finding, employers are recommended to build on their organizational capabilities to shape their personalized approach for inclusion. For instance, employers who do not see extensive possibilities to invest in extensive training and development programs, but who can offer temporary work experience jobs, may be able to contribute based on the recruitment HRM bundle. Employers, who do not have an HRM department, could focus on informal workplace practices and tailormade solutions, following the passive HRM approach.

Finally, our insights provide a steppingstone for future research into how distinct types of inclusive employers, and their different HRM approaches, can be optimally supported.

### 4.2 Strengths and limitations

The application of LCA in this study reduces the complexity of inclusive HRM bundles into a six-class model, which allows us to identify between-class differences in characteristics and groups hired. Still, our study is subject to the following four limitations.

First, to identify different inclusive employers, LCA was applied to the 2019 NEWS dataset [33] in a secondary data analysis. Although this dataset encompasses data on the most common HRM practices, these HRM practices may not be exhaustive. For instance, Chan and colleagues [29] identify other inclusion practices, such as health coverage, employee assistance programs, disability accommodation policies. Future research should explore the relevance of such practices within different contexts.

A second limitation is the cross-sectional nature of this study, which implies that we cannot draw conclusions on causality, and we cannot draw conclusions on how stable the different employer classes are over time. Investigating this stability of classes may be highly relevant for future research, as we are currently unable to estimate the impact of substantial labor market developments, such as the COVID-19 crisis [29].

Third, although our study uses a large sample of 1,665 employers, which assures enough power to estimate reliable latent profiles [48], we cannot assure generalizability of our results to all employers or all countries. Replication of this study in different samples and different countries is necessary to validate our findings.

Fourth, it should be noted that our study predicted solely the hiring of vulnerable workers, which means that we are not able to draw conclusions about the long-term employment of these vulnerable workers in their respective organizations. Future research may therefore focus on the tenure of vulnerable groups, to predict the extent to which the different classes offer sustainable employment to vulnerable groups.

## 5 Conclusion

Based on a sample of 1,665 inclusive employers, this study shows that inclusive organizations use six distinct inclusive HRM bundles, which consist of HRM practices related to the recruitment, maintenance, development, or accommodations of vulnerable workers. These inclusive HRM bundles range from extensive bundles, which consist of a multitude of these HRM practices, to more selective HRM bundles, which only consist of one or a few of these practices. With these findings, we nuance previous strategic HRM research that indicates that extensive bundling approaches are superior to individual HRM practices, by showing that not only extensive HRM bundles, but also selective HRM bundles or even passive HRM approaches are applied by organizations that successfully hire vulnerable groups. Furthermore, our results show a relationship between the application of specific bundles and the hiring of distinct groups. Hence, organizations may also tailor their HRM bundles to the needs of specific groups of vulnerable workers. As all six HRM bundles were found to contribute to inclusive employment, we recommend organization to apply a HRM bundle that fits their organizational capabilities on the one hand and the specific groups hired on the other hand to achieve a more inclusive labor market.

## Ethical approval

The data collection of NEWS-2019 was performed according to the ethical standards of the Dutch Organization for Applied Scientific Research (TNO). A full report of methodological considerations was published, and the benchmarking results of the data were made publicly available [36].

## Informed consent

All participants received an information letter before starting the questionnaire, in which the informed consent was established.

## Conflict of interest

The authors declare that they have no conflict of interest.

## Supplementary Material

Supplementary Material
