# Supplementary material for: A classification of human resource management bundles for the inclusion of vulnerable workers
Source: Work. 2024 Sep 11;79(1):177–90. doi: 10.3233/WOR-230314 (PMC11492041; doi:10.3233/WOR-230314)
Supplement: Supplementary Material [file wor-79-wor230314-s001.docx]

**Supplementary Figure 1 Visualized Item-Response Profile for the 6-Class Model of Inclusive HRM bundles**
